# Using Online Reviews by Restaurant Patrons to Identify Unreported Cases of Foodborne Illness — New York City, 2012–2013

**Published:** 2014-05-23

**Authors:** Cassandra Harrison, Mohip Jorder, Henri Stern, Faina Stavinsky, Vasudha Reddy, Heather Hanson, HaeNa Waechter, Luther Lowe, Luis Gravano, Sharon Balter

**Affiliations:** 1 New York City Department of Health and Mental Hygiene; 2 CDC/CSTE Applied Epidemiology Fellow; 3 Columbia University; 4 Yelp

While investigating an outbreak of gastrointestinal disease associated with a restaurant, the New York City Department of Health and Mental Hygiene (DOHMH) noted that patrons had reported illnesses on the business review website Yelp (http://www.yelp.com) that had not been reported to DOHMH. To explore the potential of using Yelp to identify unreported outbreaks, DOHMH worked with Columbia University and Yelp on a pilot project to prospectively identify restaurant reviews on Yelp that referred to foodborne illness. During July 1, 2012–March 31, 2013, approximately 294,000 Yelp restaurant reviews were analyzed by a software program developed for the project. The program identified 893 reviews that required further evaluation by a foodborne disease epidemiologist. Of the 893 reviews, 499 (56%) described an event consistent with foodborne illness (e.g., patrons reported diarrhea or vomiting after their meal), and 468 of those described an illness within 4 weeks of the review or did not provide a period. Only 3% of the illnesses referred to in the 468 reviews had also been reported directly to DOHMH via telephone and online systems during the same period. Closer examination determined that 129 of the 468 reviews required further investigation, resulting in telephone interviews with 27 reviewers. From those 27 interviews, three previously unreported restaurant-related outbreaks linked to 16 illnesses met DOHMH outbreak investigation criteria; environmental investigation of the three restaurants identified multiple food-handling violations. The results suggest that online restaurant reviews might help to identify unreported outbreaks of foodborne illness and restaurants with deficiencies in food handling. However, investigating reports of illness in this manner might require considerable time and resources.

## Project Protocol

Beginning in April 2012, Yelp provided DOHMH with a private data feed of New York City restaurant reviews. The feed provided data publicly available on the website but in an XML format, and text classification programs were trained to automatically analyze reviews. For this pilot project, a narrow set of criteria were chosen to identify those reviews with a high likelihood of describing foodborne illness. Reviews were assessed retrospectively, using the following criteria: 1) presence of the keywords “sick,” “vomit,” “diarrhea,” or “food poisoning” in contexts denoting foodborne illness; 2) two or more persons reported ill; and 3) an incubation period ≥10 hours. Ten hours was chosen because most foodborne illnesses are not caused by toxins but rather by organisms with an incubation period of ≥10 hours (1). Data mining software was used to train the text classification programs (2). A foodborne disease epidemiologist manually examined output results to determine whether reviews selected by text classification met the criteria for inclusion, and programs with the highest accuracy rate were incorporated into the final software used for the pilot project to analyze reviews prospectively.

The software program downloaded weekly data and provided the date of the restaurant review, a link to the review, the full review text, establishment name, establishment address, and scores for each of three outbreak criteria (i.e., keywords, number of persons ill, and incubation period), plus an average of the three criteria. Scores for individual criteria ranged from 0 to 1, with a score closer to 1 indicating the review likely met the score criteria.

Reviews submitted to Yelp during July 1, 2012–March 31, 2013 were analyzed. All reviews with an average review score of ≥0.5 were evaluated by a foodborne disease epidemiologist (Figure). Because the average review score was calculated by averaging the individual criteria scores, reviews could receive an average score of ≥0.5 without meeting all individual criteria. Reviews with an average review score of ≥0.5 were evaluated for the following three criteria: 1) consistent with foodborne illness occurring after a meal, rather than an alternative explanation for the illness keyword; 2) meal date within 4 weeks of review (or no meal date provided); 3) two or more persons ill or a single person with symptoms of scombroid poisoning or severe neurologic illness. Reviews that met all three of these criteria were then investigated further by DOHMH. In addition, reviews were investigated further if manual checking identified multiple reviews within 1 week that described recent foodborne illness at the same restaurant.

To identify previously reported complaints of foodborne illness, reviews were compared with complaints reported to DOHMH by telephone or online at 311, New York City’s nonemergency information service that can be used by the public to report suspected foodborne illness (3). Yelp reviews categorized as indicating recent or potentially recent illness were compared with complaints from the previous 4 weeks in the 311 database. To follow up with reviewers, DOHMH created a Yelp account to send private messages to reviewers’ Yelp accounts. Reviewers needed to log in at Yelp to view their messages.

For reviews not requiring further investigation and not found in the 311 database, DOHMH sent messages advising reviewers of the availability of 311 reporting. For reviews requiring further investigation, DOHMH sent messages requesting telephone interviews. Reviewers consenting to interviews were asked to provide details about the restaurant visit, meal date, foods consumed during the meal, party size, illness symptoms, and a history of foods consumed in the 3 days before symptom onset.

## Review-Based Findings

During July 1, 2012–March 31, 2013, the software system screened approximately 294,000 reviews and identified 893 with an average score of ≥0.5, indicating possible foodborne illness (Figure). Of these reviews, 499 (56%) described an event consistent with foodborne illness, as determined by the manual checking of a foodborne epidemiologist. This equated to an average of 23 reviews evaluated by a foodborne epidemiologist each week, with an average of 13 reviews categorized as consistent with foodborne illness. The remaining 394 (44%) reviews contained keywords but did not suggest foodborne illness (e.g., “I didn’t get sick at all after my meal”).

Of the 499 reviews describing an event consistent with foodborne illness, 468 (94%) indicated recent or potentially recent illness. Of these 468 reviews, only 15 (3%) were also reported to 311 during the same period. A total of 339 reviews that indicated only one person became ill and had no scombroid poisoning or severe neurologic symptoms were excluded, leaving 129 reviews that required further investigation (Figure). Of the 129, a total of 27 (21%) reviewers completed a telephone interview inquiring about meals and illnesses. The median time from review date to DOHMH contact to schedule a telephone interview was 8 days. The interviews provided information on 27 restaurants, and 24 restaurants were identified as potential locations of recent exposure because the meal dates were within 4 weeks of the interview.

From the 27 interviews, DOHMH determined whether the complaints warranted an outbreak investigation by considering the following criteria: 1) more than one person became ill, 2) no other common meals were suspected, 3) ill persons lived in different households, and 4) the cases had similar onset periods (indicating a likely foodborne cause rather than person-to-person transmission). For scombroid poisoning or neurologic symptoms, DOHMH considered whether symptoms and onset were consistent with scombrotoxin, ciguatera toxin, or botulism poisoning.

Three outbreaks meeting DOHMH outbreak investigation criteria were identified, accounting for 16 illnesses not previously reported to DOHMH. Interviews with reviewers identified likely food items associated with illness at each of the three restaurants: house salad, shrimp and lobster cannelloni, and macaroni and cheese spring rolls (Table). The reviews of the three restaurants had been posted on Yelp 2–5 days after the meals. Environmental investigations were conducted at two of the three restaurants during the week after the interviews; a routine DOHMH inspection had already been conducted at the other restaurant 2 days after the meal. The two investigations and the routine inspection identified multiple violations at each of the outbreak restaurants (Table). Investigators were unable to obtain laboratory data that might have identified the infectious agents.

### Discussion

In a New York City DOHMH pilot project, of 468 recent or potentially recent online foodborne illness complaints posted on Yelp and reviewed by foodborne epidemiologists, three previously unreported restaurant outbreaks were identified. Because foodborne cases have a common exposure, a restaurant patron review-based system can identify small, point-source outbreaks that are not easily found by systems reviewing large sources of data, such as syndromic surveillance of emergency department visits (4), Google Flu Trends (5), and analysis of Twitter data for influenza and other public health trends (6–8). Most importantly, foodborne epidemiologists can confirm reports because Yelp offers a way to follow-up with reviewers for interview.

In this project, only 15 (3%) of the 468 recent or potentially recent illnesses identified on Yelp were also reported directly to New York City’s nonemergency 311 service, suggesting that knowledge about 311 reporting is limited. Of further note, after messages regarding the availability of 311 were sent to 290 reviewers who did not meet the project criteria, 32 responded, of whom 25 (78%) said they were unaware of the 311 system or would keep 311 in mind for the future. The 311 service receives approximately 3,000 food poisoning complaints each year, and from that number, about 1% are identified as outbreak-related (DOHMH, unpublished data, 2014).

As social media usage continues to grow among U.S. adults (9), health departments might consider additional surveillance methods to capture illness reports from those more likely to post a restaurant review online than to contact a health department. By incorporating website review data into public health surveillance programs, health departments might find additional illnesses and improve detection of foodborne disease outbreaks in the community. Similar programs could be developed to identify other public health hazards that reviewers might describe, such as vermin in food establishments.

The findings in this report are subject to at least four limitations. First, to increase the likelihood of identifying true foodborne illness, a narrow focus was chosen for the individual criteria used to score reviews. Therefore, it is possible that some foodborne illnesses were not picked up by the screening software because of low average review scores (e.g., because of illnesses resulting from toxins with short incubation periods). Second, personal contact information for reviewers was unavailable, requiring reviewers to check their Yelp accounts and provide a telephone number to participate, which extended the time from review to interview and might have affected the response rate. Third, investigators were not able to identify any of the infectious agents in the outbreaks. Finally, the system required substantial resources; in addition to programming expertise, staff members were needed to read reviews, send e-mails, interview reviewers, and perform follow-up inspections.

What is already known on this topic?

Health departments rely on the public to report restaurant-related foodborne illness directly to them, yet many outbreaks go unreported. A large amount of publicly reported information about foodborne illness is available on restaurant review websites.

What is added by this report?

During a 9-month period, approximately 294,000 reviews of New York City restaurants posted on Yelp.com were screened by software programs for possible cases of foodborne illness. The software flagged 893 reviews for evaluation by an epidemiologist, resulting in the identification of 468 reviews that were consistent with recent or potentially recent foodborne illness. Only 15 (3%) of these reviews described events that had been reported to the health department. After further evaluation of reviews and interviews with 27 reviewers, three previously unreported restaurant-related outbreaks were identified.

What are the implications for public health practice?

Review websites might be a valuable source of data in the public health setting. Restaurant patron reviews can help identify small, point-source outbreaks of foodborne illness because cases have a known common exposure. Such reviews might be particularly useful if the website offers a way to reach reviewers for follow-up interviews.

Additional work using social media might improve health department abilities to use the Internet for disease detection. Working with the Chicago Department of Public Health, the Smart Chicago Collaborative recently developed a system to contact those who post foodborne illness complaints either on its website or on Twitter.* For health departments looking for an alternative to analyzing review data weekly, creating an illness-reporting vehicle such as the Utah Department of Health’s “I Got Sick” website (10) could be a more practical solution, although it might be less widely used than a review website such as Yelp. Review websites could assist by offering a link to the reviewer’s local health department’s reporting system at the time of review posting.

DOHMH plans to continue to refine this project. To shorten the time from review to investigation, Yelp will provide daily instead of weekly review feeds, and, to increase sensitivity, the project will be expanded to include additional review websites. To improve response rates, DOHMH will offer a link to an electronic survey. Finally, DOHMH is exploring the possibility of linking multiple complaints pertaining to the same restaurant, using data from different review websites and DOHMH databases.

## Figures and Tables

**FIGURE:**
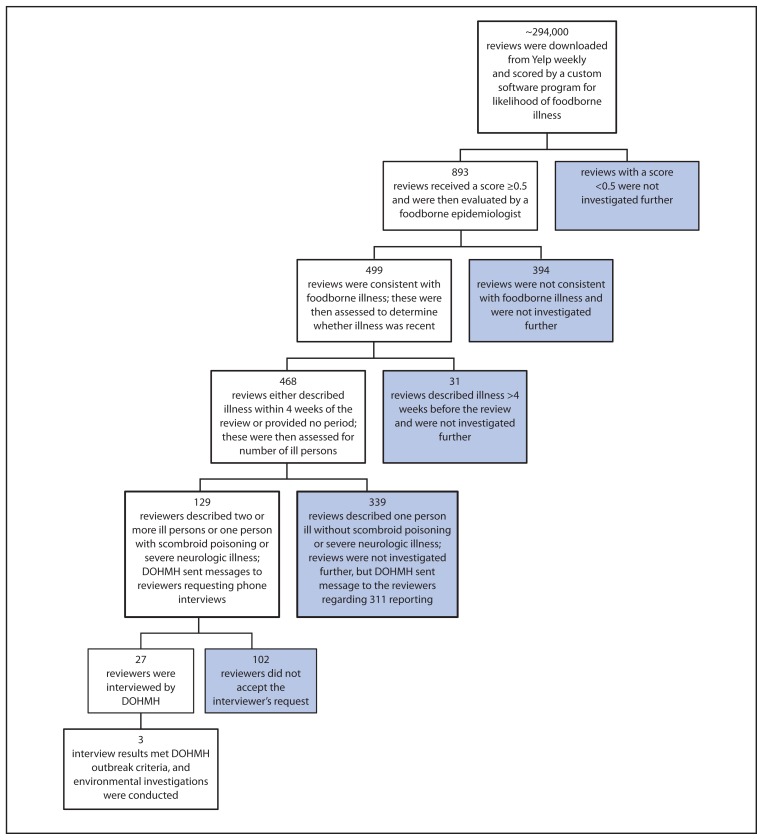
Results of investigation of online reviews by restaurant patrons that referred to possible foodborne illness — pilot project, New York City, July 1, 2012–March 31, 2013 **Abbreviation:** DOHMH = Department of Health and Mental Hygiene.

**TABLE:** Unreported outbreaks of foodborne illness identified by investigation of online restaurant patron reviews — pilot project, New York City, July 1, 2012–March 31, 2013

Outbreak	Month of meal	Likely food vehicle	No. of persons ill/No. in reviewer’s party	Public health action	Environmental findings
Outbreak A	December 2012	House salad	7/9	Environmental investigation and food preparation review conducted in response to interview with reviewer	Cross-contamination in refrigerator Bare-hand contact with ready-to-eat food Improperly sanitized work surfaces No washing of ready-to-eat vegetables
Outbreak B	January 2013	Shrimp and lobster cannelloni	3/5	Routine inspection conducted 2 days after meal	Improper cold food storage Improper thawing procedures Food contact surface not maintained properly Food dispensing utensils stored improperly Mouse activity present Live roaches present
Outbreak C	March 2013	Macaroni and cheese spring rolls	6/6	Environmental investigation and food preparation review conducted in response to interview with reviewer	Bare-hand contact with ready-to-eat food Cold storage temperatures not taken during cold holding of pre-prepared food

## Footnotes

* Available at https://foodborne.smartchicagoapps.org.
